# Porcine Rotavirus NSP4 Inhibits Type I Interferon Production via NRBF2‐Mediated Autophagic Degradation of MDA‐5

**DOI:** 10.1155/tbed/5789277

**Published:** 2026-06-24

**Authors:** Jiaxing Zhan, Tianhao Liang, Jiale Chen, Jingying Wang, Enqi Dai, Ting Wang, Shaojie Wang, Zixuan Cao, Hanwei Jiao, Yan Zeng, Chunfeng Wang, Xin Cao

**Affiliations:** ^1^ College of Veterinary Medicine, Jilin Agricultural University, Changchun, 130118, China, jlau.edu.cn; ^2^ Engineering Research Center of Microecological Vaccines (Drugs) for Major Animal, Jilin Agricultural University, Changchun, 130118, China, jlau.edu.cn; ^3^ Diseases, Ministry of Education, Jilin Agricultural University, Changchun, 130118, China, jlau.edu.cn; ^4^ The College of Veterinary Medicine, Southwest University, Chongqing, 402460, China, swu.edu.cn

**Keywords:** autophagy, IFN-I, MDA-5, NRBF2, PoRV-NSP4

## Abstract

The type I interferon (IFN‐I) signaling pathway plays a pivotal role in orchestrating antiviral innate immune defenses, particularly during the clearance of invading pathogens. Rotaviruses have evolved a repertoire of viral proteins to counteract host immune surveillance. While certain functions of rotavirus nonstructural proteins in antagonizing IFN‐I signaling have been characterized, the precise molecular mechanism by which nonstructural protein 4 (NSP4) impairs host immunity remains elusive. Here, we demonstrated that the porcine rotavirus (PoRV) nonstructural protein NSP4 potently suppresses the transcriptional activation of interferon‐stimulated genes (ISGs), IFN‐β promoters, and interferon‐sensitive response elements (ISREs) while abrogating the phosphorylation of interferon regulatory factor 3 (IRF3). Mechanistically, NSP4 promotes the degradation of melanoma differentiation‐associated gene 5 (MDA‐5) via nuclear receptor binding factor 2 (NRBF2)‐dependent autophagy, thereby subverting IFN‐I production. The validity of this mechanism in primary epithelial cells was also verified by constructing an intestinal organoid model in piglets. Collectively, our findings elucidate a previously unrecognized immune evasion mechanism by which NSP4 antagonizes the IFN‐I‐mediated antiviral response, providing novel molecular insights for developing therapeutic strategies against rotavirus infections.

## 1. Introduction

Porcine rotavirus (PoRV), a member of the Reoviridae family within the *Rotavirus* genus, is a nonenveloped, double‐stranded RNA virus that constitutes a major etiological agent of severe acute gastroenteritis in neonatal piglets [1]. As a highly infectious enteric pathogen with zoonotic potential, PoRV poses a significant threat to public health security and inflicts substantial economic losses on the global swine industry [2]. Its genome comprises 11 segments of dsRNA encoding six structural proteins (VP1–VP4, VP6, and VP7) and six nonstructural proteins (NSP1–NSP6) [3, 4]. These viral components orchestrate a coordinated strategy to facilitate viral replication and particle assembly while simultaneously subverting host immune defenses through multifaceted virus–host interactions. Notably, nonstructural proteins exhibit sophisticated immune evasion mechanisms that synergistically counteract intracellular antiviral surveillance pathways [5].

Among the nonstructural proteins of PoRV, nonstructural protein 4 (NSP4) has garnered significant attention because of its functional versatility. As the first identified viral enterotoxin, NSP4 exhibits viroporin activity by forming virus‐encoded ion channels [6] that mediate endoplasmic reticulum (ER) calcium efflux into the cytosol, thereby disrupting cellular calcium homeostasis [7, 8]. This dysregulation of intracellular Ca^2+^ triggers pathophysiological consequences, including secretory diarrhea, by inducing excessive electrolyte and fluid secretion in intestinal epithelial cells—a hallmark pathology of RV infection [9]. Concomitantly, NSP4‐driven calcium signaling activates the calcium/calmodulin‐dependent kinase CAMKK2 and the downstream AMPK pathway, initiating autophagy, which critically supports both viral particle maturation and replication [10]. Furthermore, the RV NSP4 orchestrates ER‐localized virion assembly and egress through coordinated interactions with autophagy adaptors LC3, effectively hijacking the host machinery for viral propagation [11].

Indeed, previous literature suggests that secreted NSP4, by inducing calcium fluxes, activates the innate immune defenses of the host by acting as a pathogen‐associated molecular pattern (PAMP) that stimulates TLR2 on macrophages with downstream activation of NF‐κB and subsequent release of proinflammatory cytokines [12]. Rotaviruses have two other proteins (VP3 and NSP1) that effectively downregulate the innate immune defenses of infected enterocytes. The VP3 protein exploits the host ubiquitin‐proteasome machinery to induce proteasomal degradation of mitochondrial antiviral‐signaling protein (MAVS), thereby abolishing IFN‐III production [13]. In addition, through a proteasome‐dependent mechanism, NSP1 simultaneously targets interferon regulatory factor 3 (IRF3), IRF5, IRF7, and TNF receptor‐associated factor 2 (TRAF2) for degradation, resulting in coordinated suppression of both type I interferon (IFN‐I) synthesis and NF‐κB signaling activation [14–16].

While the roles of NSP4 in calcium homeostasis, autophagy regulation, and virion assembly have been extensively characterized [17], its potential function in host immune evasion remains poorly defined. Cellular antiviral defenses are initiated when pattern recognition receptors (PRRs), such as RIG‐I and melanoma differentiation‐associated gene 5 (MDA‐5), detect viral RNA [18], triggering downstream signaling cascades that activate IFN‐I production to suppress viral replication [19]. Notably, PoRV employs multiple countermeasures to subvert this immune response [20].

This study reveals the critical role of PoRV‐NSP4 in immune evasion. We demonstrated that NSP4 recruits nuclear receptor binding factor 2 (NRBF2) to hijack the autophagic pathway, specifically mediating degradation of the PRR MDA‐5, thereby markedly suppressing IFN‐I production. This mechanism identifies NSP4 not only as a key player in viral replication but also as a central orchestrator of immune evasion. Our findings deepen the understanding of the immune escape strategies of PoRV and provide a theoretical foundation for developing NSP4‐targeted antiviral interventions while offering insights into designing safer and more efficacious rotavirus vaccines.

## 2. Materials and Methods

### 2.1. Cells and Viruses

The HEK293T cell line (derived from human embryonic kidney epithelial cells; ATCC, Catalog Number CRL‐3216), LLC‐PK1 cell line (derived from porcine kidney epithelial cells; ATCC, Catalog Number bio‐131297) were cultured in Dulbecco’s modified Eagle’s medium (Gibco, C11995500BT) supplemented with 10% fetal bovine serum (FBS) (Gibco, 16000069) at 37°C under 5% CO_2_. PoRV strain DN30209 was kindly provided by Professor Xiaofeng Ren [21]. SeV was stored in our laboratory.

### 2.2. Antibodies and Reagents

#### 2.2.1. Antibodies

Anti‐β‐actin antibody (A2228), anti‐Flag agarose beads (A2220), and anti‐HA agarose beads (A2095) were purchased from Sigma–Aldrich. Horseradish peroxidase (HRP)‐conjugated mouse secondary antibodies (anti‐HA‐HRP, anti‐Flag‐HRP) (130‐091‐972; AF2855), DMSO (ST038), Alexa Fluor 488‐conjugated goat anti‐rabbit antibodies (A0423), and Alexa Fluor 647‐conjugated goat anti‐mouse antibodies (A0473) were obtained from Beyotime Biotechnology Co., Ltd. Anti‐IRF3 (4302) and anti‐phospho‐IRF3 (p‐IRF3) (4947) antibodies were procured from Cell Signaling Technology. HRP‐conjugated goat anti‐mouse IgG (H+L) (SA00001‐1), goat anti‐rabbit IgG (H+L) (SA00001‐2), anti‐MDA‐5 (21775‐1‐AP), anti‐RIG‐I (25068‐1‐AP), anti‐LC3 (14600‐1‐AP), anti‐P62 (18420‐1‐AP), and anti‐MAVS (14341‐1‐AP) endogenous antibodies were obtained from Proteintech Group, Inc. The anti‐NSP4 polyclonal antibody was generated by Jieyu Biotechnology Co., Ltd. The NRBF2 antibody (sc‐365213) was obtained from Santa Cruz Biotechnology, Inc. The anti‐PoRV‐VP6 polyclonal antibody was prepared in our laboratory.

#### 2.2.2. Reagents

RIPA lysis buffer (HY‐K1001), 3‐methyladenine (3‐MA) (HY‐19312), Z‐VAD‐FMK (Z‐VAD) (HY‐16658B), chloroquine (CQ) (HY‐17589A), and Poly(I:C) (HY‐107202) were purchased from MedChemExpress. Moloney murine leukemia virus (M‐MLV) reverse transcriptase (M1701) was obtained from Promega Corporation. The SYBR Green mix (Q312), TRIzol (R411), Lipomaster 2000 Transfection Reagent (TL201), and the Lipofectamine 3000 (TL301) were obtained from Vazyme. The Dual‐Luciferase Reporter Assay Kit (RG028) and Lipo6000 (C0526) were obtained from Beyotime Biotechnology Co., Ltd. A plasmid DNA purification kit (D6943) and FBS (FB‐03) were purchased from Omega Bio‐Tek. Polyvinylidene fluoride (PVDF) (03010040001) membranes were procured from Merck Millipore. Triton X‐100 (T8200) and phosphate‐buffered saline (PBS) (P1020) were obtained from Solarbio Life Sciences Co., Ltd. Corning Matrigel (354234) was procured from CORNING, and IntestiCult Intestinal Organoid Differentiation Medium (100‐0214) was procured from STEMCELL.

### 2.3. Construction and Transfection of the Plasmids

The NSP4 gene of PoRV (GenBank: KC190292.1) was synthesized and cloned, and inserted into pRK‐HA and pRK‐Flag vectors using standard molecular biology techniques at SalI and NotI restriction enzyme sites. Plasmid DNA from recombinant *E. coli* was purified using an endotoxin‐free plasmid extraction kit according to the manufacturer’s protocol. The IFN‐β‐Luc and ISRE‐Luc luciferase reporter plasmids have been described previously. The plasmids pCMV‐NRBF2, pCMV‐AKT2, and pCMV‐DNM2 were synthesized by GENEWIZ Biotech Co., Ltd. The plasmids pRK‐Flag‐Myd88, pRK‐Flag‐TRIF, pRK‐Flag‐RIG‐I, pRK‐Flag‐RIG‐IN, pRK‐Flag‐MAVS, pRK‐Flag‐MDA‐5‐N, pRK‐Flag‐TRAF3, pRK‐Flag‐TBK1, pRK‐Flag‐IKKi, and pRK‐Flag‐IRF3 were maintained in our laboratory. For transient transfection, purified plasmids were transfected into LLC‐PK1 cells using Lipofectamine 3000 (plasmid‐to‐reagent ratio 1:3) or into HEK293T cells using Lipo6000 (plasmid‐to‐reagent ratio 1:2), followed by incubation in complete DMEM supplemented with 10% FBS at 37°C for 24 h prior to subsequent experiments.

### 2.4. RT‐PCR

Total RNA was isolated from samples using TRIzol reagent. First‐strand cDNA was synthesized from 1 μg of total RNA with M‐MLV reverse transcriptase by incubation at 80°C for 10 min and 37°C for 2 h, followed by storage at 4°C for immediate use or −20°C for long‐term preservation. Real‐time quantitative PCR was performed using SYBR Green Mix on an Applied Biosystems QuantStudio 5 Real‐Time PCR System, following the manufacturer’s protocols. The relative mRNA expression levels of the target genes were calculated using the 2^−ΔΔCt^ method, with the data normalized to the housekeeping gene GAPDH. The sequences of primers used in this study are listed in Table 1.

**Table 1:** The primer sequences for RT‐qPCR.

Primers	Sequence (5′→3′)
Pig IFIT2‐forward	AAGCACCTCAAAGGGCAAAAC
Pig IFIT2‐reverse	TCGGCCCATGTGATAGTAGAC
Pig CXCL10‐forward	GTGGCATTCAAGGAGTACCTC
Pig CXCL10‐reverse	TGATGGCCTTCGATTCTGGATT
Pig ISG15‐forward	GATCGGTGTGCCTGCCTTC
Pig ISG15‐reverse	CGTTGCTGCGACCCTTGT
Pig ISG56‐forward	AAATGAATGAAGCCCTGGAGTATT
Pig ISG56‐reverse	AGGGATCAAGTCCCACAGATTTT
Pig OAS1‐forward	GCCTGTGATTCTGGACCCGGCTGA
Pig OAS1‐reverse	CGACACCTTCCAGGATCCCACCG
Pig IFN‐α‐forward	CTGCTGCCTGGAATGAGAGCC
Pig IFN‐α‐reverse	TGACACAGGCTTCCAGGTCCC
Pig IFN‐β‐forward	GCTAACAAGTGCATCCTCCAAA
Pig IFN‐β‐reverse	AGCACATCATAGCTCATGGAAAGA
Pig IFN‐λ1‐forward	ACTGTGATGCTGGACTTGG
Pig IFN‐λ1‐reverse	GCATCCTTGGCTTTCTTGAAG
Pig IFN‐λ3‐forward	ACTTGGCCCAGTTCAAGTCT
Pig IFN‐λ3‐reverse	CATCCTTGGCCCTCTTGA
Pig GAPDH‐forward	ACATGGCCTCCAAGGAGTAAGA
Pig GAPDH‐reverse	GATCGAGTTGGGGCTGTGACT

### 2.5. siNSP4‐Mediated Knockdown

The NSP4 siRNA primers used are listed in Table 2. For siRNA transfection, plasmids were transfected into LLC‐PK1 cells using Lipofectamine 2000 (plasmid‐to‐reagent ratio 1:3) for 24 h. Subsequently, the cells were infected with PoRV at a multiplicity of infection (MOI) of 1 for 6‐, 12‐, or 24‐h durations. The mRNA levels of IFN‐α, IFN‐β, IFN‐λ, ISG15, ISG56, and OAS1 were quantified via RT‐PCR. The protein expression levels of IRF3 and p‐IRF3 were assessed by Western blotting.

**Table 2:** siRNA sequences used in this study.

Primers	Sequence (5′→3′)
siNSP4‐forward	GAUGAGACGUCAACUGGAAAUGAUU/dT//dT/
siNSP4‐reverse	AAUCAUUUCCAGUUGACGUCUCAUC/dA//dT/
siNRBF2‐forward	GGCUCAUUUAUCACUGGAA/dT//dT/
siNRBF2‐reverse	UUCCAGUGAUAAAUGAGCC/dT//IdT/
siNC‐forward	UUCUCCGAACGUGUCACGU/dT//dT/
siNC‐reverse	ACGUGACACGUUCGGAGAA/dT//dT/

### 2.6. Western Blotting

The desired plasmids were transfected into HEK293T or LLC‐PK1 cells. After 24–48 h of transfection, the culture medium was removed, and the cells were lysed with RIPA buffer. Total cellular proteins were collected, mixed with 2× SDS loading buffer, and boiled for 15 min at 95°C. Proteins were resolved by SDS–PAGE (8%–12.5% gradient gels) and electrophoretically transferred onto methanol‐activated PVDF membranes. The membranes were blocked with 5% skim milk in TBST for 1 h at room temperature with gentle agitation, followed by overnight incubation with primary antibodies at 4°C. After being washed with PBST (0.5% Tween‐20), the membranes were incubated with HRP‐conjugated secondary antibodies for 1 h at room temperature. The protein bands were visualized using an Amersham Imager 600 RGB system (Cytiva) after additional PBST washes.

### 2.7. Dual‐Luciferase Reporter Assays

HEK293T cells were cotransfected with Lipofectamine 3000 for 24 h using plasmids encoding Flag‐tagged RIG‐I, MAVS, MDA‐5, TBK1, IKKε, or HA‐tagged NSP4, along with IFN‐β‐Luc, ISRE‐Luc reporter plasmids, and the internal control pRL‐TK plasmid. The cells were harvested at specified time points, and dual‐luciferase activity was measured using the Dual‐Luciferase Reporter Assay System according to the manufacturer’s protocol.

### 2.8. Indirect Immunofluorescence Assay (IIFA)

The cells were seeded on poly‐L‐lysine (0.1%)‐coated coverslips placed in culture dishes. After 24 h of transfection, the samples were collected and rinsed once with PBS. The cells were fixed with 4% paraformaldehyde for 15 min, washed again with PBS, and blocked with PBS containing 0.5% Triton X‐100 and 5% skim milk at 4°C for 12 h. Following blocking, the cells were incubated overnight at 4°C with primary antibodies (derived from different species). After being washed with PBS, the cells were incubated with fluorophore‐conjugated secondary antibodies at room temperature for 1 h, followed by washing with PBS. Nuclei were stained with DAPI (2.5 µg/mL) for 5 min, washed with PBS, and mounted with antifade mounting medium. The images were observed using an LSM 510 Zeiss confocal microscope (Carl Zeiss Jena).

### 2.9. Coimmunoprecipitation (Co‐IP)

HEK293T cells were cotransfected with the target plasmids. At 24 h posttransfection, the cells were harvested and lysed with NP‐40 lysis buffer. For each IP reaction, 1 mL of lysate was incubated with anti‐Flag/HA agarose affinity gel under gentle rotation at 4°C for 12 h. Beads were washed three times with NP‐40 lysis buffer and eluted by boiling in 2× SDS loading buffer at 95°C for 15 min. Immunoprecipitated proteins were subjected to Western blotting using anti‐Flag and anti‐HA antibodies.

### 2.10. GST‐Tagged Protein Purification

Primers F (5′‐CCGGAATTCGCCACCATGGCTTCAGGCATCCTGGA‐3′) and R (5′‐CCGCTCGAGTAGAGCCCGGCGAGGAGCGAGAGAGCACAGAA‐3′), containing NotI and SalI cleavage sites, respectively, were designed and synthesized by Sangon Biotech, Shanghai, China. Isopropyl β‐d‐1‐thiogalactopyranoside (IPTG) was used to induce the NSP4‐GST fusion protein, and the purification of the NSP4 protein was carried out according to the methods of our laboratory.

### 2.11. GST Pull‐Down

Purified GST‐tagged NSP4 and GST‐only control protein (150 µg each) were individually mixed with 100 µL of glutathione‐Sepharose beads in microcentrifuge tubes and incubated at 4°C for 2 h with gentle rotation. The beads were washed three times with PBS containing 1% Triton X‐100, followed by three additional washes with PBS alone. A 50 µL aliquot of each sample was retained to verify the GST‐protein binding efficiency via SDS–PAGE. To the remaining bead‐bound proteins, 150 µg of total protein lysate from porcine IPEC was added, and the total volume was adjusted to 600 µL with PBS, followed by incubation at 4°C for 2 h. After repeated washes, the proteins were eluted by boiling in 1× SDS loading buffer at 100°C for 10 min, centrifuged at 12,000 × g for 2 min, and analyzed by SDS–PAGE.

### 2.12. Liquid Chromatography‐Tandem Mass Spectrometry (LC‐MS/MS) Analysis and Prediction

Protein bands showing differential expression (compared with the control group) were excised from gels and subjected to LC–MS/MS analysis using a Q Exactive Hybrid Quadrupole‐Orbitrap mass spectrometer (Thermo Fisher Scientific, USA). The raw data files were processed with MaxQuant software (v1.5.5.1) for peptide identification and matched against the UniProt/Swiss‐Prot database for species‐specific protein annotation. Kyoto Encyclopedia of Genes and Genomes (KEGG) enrichment analysis was performed using the OmicShare tool (https://www.omicshare.com/tools/home/report/koenrich.html).

### 2.13. Organoid Extraction and Culture

According to the American Veterinary Medical Association veterinary guidelines, euthanasia is performed by administering sodium pentobarbital to anesthetize piglets, followed by cutting the carotid artery to bleed them to death. The organoid extraction and culture were carried out as described by Wang et al. [22].

### 2.14. Protein Structure Prediction and Modeling

The three‐dimensional structures of NSP4, NRBF2, and MDA‐5 were predicted using AlphaFold3 (https://AlphaFold.ebi.ac.uk/) with default parameters. The structural alignment and interaction interfaces between NSP4, NRBF2, and MDA‐5 were analyzed using the PyMOL Molecular Graphics System (v2.5.4) (https://pymol.org/).

### 2.15. Statistical Analysis

All samples were analyzed using GraphPad Prism 8 software. The data were analyzed by an unpaired two‐tailed Student’s *t* test or two‐way ANOVA with Sidak’s test to correct for multiple comparisons. The differences are considered statistically significant at  ^∗^ *p*  < 0.05,  ^∗∗^ *p*  < 0.01, and  ^∗∗∗^ *p*  < 0.001.

## 3. Results

### 3.1. PoRV NSP4 Inhibits the Activation of Interferon‐Stimulated Genes (ISGs) and IRF3

**Figure 1:**
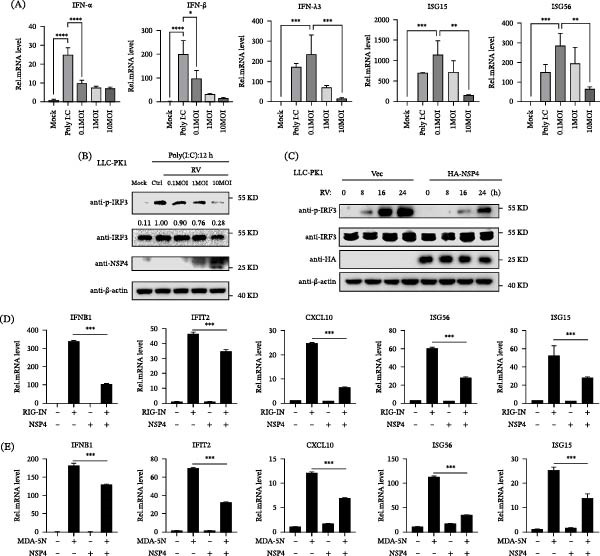
PoRV NSP4 inhibits the activation of ISGs and IRF3. (A) LLC‐PK1 cells were infected with RV at different doses for 12 h, followed by transfection with Poly(I:C). The transcriptional levels of ISGs were then detected by qRT‐PCR. (B) LLC‐PK1 cells were infected with RV at different doses for 12 h, followed by transfection with Poly(I:C). IRF3 phosphorylation was analyzed by Western blotting. (C) Western blotting was used to detect the effect of NSP4 on p‐IRF3 in PoRV‐infected LLC‐PK1 cells at various time points. (D, E) RIG‐IN (500 ng) and MDA‐5N (500 ng) plasmids were transfected into HEK293T cells to activate the IFN‐I pathway, along with NSP4 (1 μg) or empty vector plasmids. Twenty‐four hours after transfection, the mRNA expression of the IFN‐I‐stimulated genes IFNB1, ISG56, IFIT2, CXCL10, and ISG15 was determined by RT‐PCR. All the experiments were independently repeated at least three times. The data are expressed as the means ± standard deviations; *n* = 3. *p* < 0.05 were considered statistically significant ( ^∗^ *p*  < 0.05,  ^∗∗^ *p*  < 0.01,  ^∗∗∗^ *p*  < 0.001, and  ^∗∗∗∗^ *p*  < 0.0001).

To determine whether RV suppresses IFN‐I signaling, we examined the effect of RV infection on Poly(I:C)‐ or SeV‐induced IFN‐I responses. RV infection markedly reduced the transcription of IFN‐α, IFN‐β, OAS1, IFN‐λ3, ISG15, and ISG56 induced by Poly(I:C) or SeV (Figure 1A and Supporting Information 1: Figure S1A) and inhibited IRF3 phosphorylation (Figure 1B and Supporting Information 1: Figure S1B,C), indicating that RV antagonizes canonical IFN‐I signaling activation. Furthermore, NSP4 overexpression in LLC‐PK1 cells markedly decreased p‐IRF3 levels. To determine whether NSP4 regulates key components of the IFN‐I pathway, we measured the transcriptional levels of IFNB1, ISG56, IFIT2, CXCL10, and ISG15. RIG‐IN and MDA‐5‐N robustly activated the IFN‐I pathway, whereas NSP4 overexpression significantly suppressed the upregulation of these ISGs (Figure 1D,E). These results suggest that PoRV‐NSP4 inhibits IRF3 activation by targeting the RLR signaling axis, thereby attenuating IFN‐I‐mediated antiviral responses.

### 3.2. NSP4 Acts Upstream of MAVS to Degrade MDA‐5 via the Autophagy Pathway

To determine the functional site of NSP4 within the RLR signaling pathway, the impact of NSP4 on RIG‐IN, MDA‐5‐N, MAVS, TBK1, and IKKi in activating IFN‐β and interferon‐sensitive response element (ISRE) activity was assessed using a dual‐luciferase reporter assay. The results revealed that PoRV‐NSP4 significantly suppressed the activation of the IFN‐β and ISRE promoters mediated by RIG‐IN and MDA‐5‐N, whereas it had no inhibitory effect on the IFN‐β or ISRE promoter activity triggered by MAVS or its downstream components (Figure 2A). These findings indicate that PoRV‐NSP4 effectively suppresses IFN‐I responses by targeting signaling components upstream of MAVS. To confirm whether NSP4 acts on a signaling molecule upstream of MAVS, key components of the RLR pathway were cotransfected with PoRV‐NSP4 into HEK293T cells for Co‐IP assays. The results demonstrated that NSP4 specifically interacted with MDA‐5 (Figure 2B and Supporting Information 2: Figure S2A). Moreover, endogenous interaction between NSP4 and MDA‐5 was detected in PoRV‐infected LLC‐PK1 cells (Figure 2C and Supporting Information 2: Figure S2B). Structural modeling using the AlphaFold3 online tool and PyMOL revealed that NSP4 resides in the lumen of MDA‐5, where it forms a complex in which NSP4 appears enveloped by the MDA‐5 structure (Figure 2D). Indirect immunofluorescence further confirmed the spatial colocalization of NSP4 and MDA‐5 (Figure 2E). To determine whether NSP4 affects the stability of MDA‐5, a Western blotting assay was performed, which revealed that NSP4 affects the stability of MDA‐5 but not the stability of RIG‐I or MAVS in a dose‐dependent manner (Figure 2F and Supporting Information 2: Figure S2C,D). Consistently, MDA‐5 expression was progressively reduced in LLC‐PK1 cells following RV infection (Figure 2G). To identify the degradation pathway, HEK293T cells coexpressing NSP4 and MDA‐5 were treated with inhibitors (3‐MA, CQ, and Z‐VAD), and PBS and DMSO were used as controls; these treatments were applied 6 h prior to sample collection. Notably, 3‐MA and CQ effectively reversed NSP4‐mediated MDA‐5 degradation (Figure 2H), suggesting that NSP4 promotes MDA‐5 degradation via the autophagy pathway.

Figure 2NSP4 acts upstream of MAVS to degrade MDA‐5 via the autophagy pathway. (A) RIG‐IN (200 ng), MDA‐5 N (200 ng), MAVS (200 ng), TBK1 (200 ng), and IKKi (200 ng) NSP4 (300 ng) plasmids or empty vectors and ISRE and IFN‐β dual luciferase reporter plasmids were transfected into HEK293T cells to determine the effects of NSP4 on ISRE and IFN‐β promoter activity. (B) NSP4 interaction with RLR signaling molecules was detected by Co‐IP in HEK293T cells. (C) Endogenous interaction between NSP4 and MDA‐5 was examined in RV‐infected LLC‐PK1 cells. (D) NSP4 and MDA‐5 protein structures were predicted using AlphaFold3, and an interaction model was constructed using PyMOL. (E) LLC‐PK1 cells were infected with RV for 24 h, then immunostained with an anti‐MDA‐5 or anti‐NSP4 antibody as the primary antibody followed by a fluorescently labeled secondary antibody; nuclei were counterstained with DAPI. Scale bar, 10 μm. (F) Effects of NSP4 on the stability of MDA‐5 were detected by Western blotting. (G) LLC‐PK1 cells were infected with RV, and the protein levels of endogenous MDA‐5 and viral NSP4 were examined by Western blotting. (H) NSP4 and MDA‐5 plasmids were transfected into HEK293T cells for 18 h, which were then treated with ZVAD (20 µM), 3‐MA (10 mM), 3‐MA (20 mM), CQ (20 mM), DMSO, or PBS for 6 h, followed by Western blotting. All the experiments were independently repeated at least three times. The data are expressed as the means ± standard deviations; *n* = 3. *p* < 0.05 were considered statistically significant ( ^∗^ *p*  < 0.05,  ^∗∗^ *p*  < 0.01,  ^∗∗∗^ *p*  < 0.001, and  ^∗∗∗∗^ *p*  < 0.0001).

**Figure:**
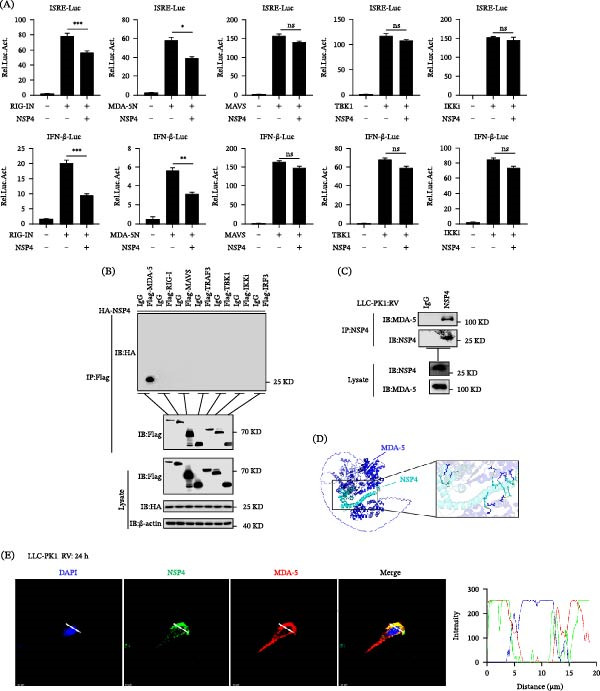

**Figure:**
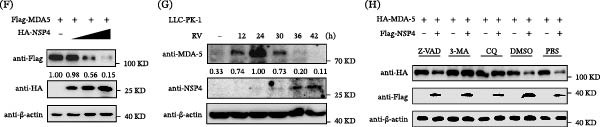

### 3.3. NSP4 Interacts With the Host Protein NRBF2

To investigate the direct interaction between NSP4 and MDA‐5 in vivo, we generated pGEX‐4T‐1‐NSP4 (47‐175) recombinant plasmids and achieved successful protein expression and purification, as confirmed by electrophoretic analysis (Supporting Information 1: Figure S1A). Following the validation of protein integrity, GST pull‐down assays were performed by incubating NSP4 with total cellular lysates from PoRV‐infected IPEC‐J2 cells. The putative host interactors captured by NSP4 (Supporting Information 1: Figure S1B) were subjected to LC‐MS/MS for identification and quantitative profiling (Figure 3A). Among the 30 high‐confidence NSP4‐interacting host proteins identified (Figure 3B), no detectable association with MDA‐5 was detected (Figure 3C). KEGG pathway analysis revealed that these proteins were functionally enriched in viral infectious diseases (Figure 3D). Notably, significant pathway enrichment was detected in the autophagy‐animal signaling pathway (Figure 3E). We reasoned that NSP4 modulates the stability of MDA‐5 via the autophagy pathway; therefore, we focused on two autophagy‐related host proteins identified in our pull‐down assay, AKT2 and NRBF2 (Supporting Information 1: Figure S1C). AKT2 is known to suppress autophagy initiation and the overall autophagic flux [23, 24]. NRBF2 was annotated as a core component of the class III phosphatidylinositol 3‐kinase (PI3KC3) complex, a critical regulator of autophagy initiation [25]. This finding aligns with prior reports demonstrating NSP4‐induced autophagic flux [11], suggesting that autophagy‐mediated degradation may represent a principal mechanism through which NSP4 suppresses IFN‐I responses, potentially involving NRBF2‐dependent pathways. To further characterize the functional linkage between NSP4 and NRBF2, we performed immunofluorescence colocalization assays to examine the spatial relationships between NSP4 and the candidate host proteins identified by pull‐down. Among the screened interactors, NRBF2 and AKT‐2 exhibited substantial subcellular codistribution with NSP4 (Figure 3F). Subsequent Co‐IP validation revealed no physical interaction between NSP4 and DNM2/AKT‐2 (Figure 3G,H), whereas robust binding affinity was observed between NSP4 and NRBF2 (Supporting Information 3: Figure S3E,F). Similar results were also observed under infection conditions (Figure 3I and Supporting Information 3: Figure S3D). Structural analysis of the predicted three‐dimensional model of the PoRV‐NSP4, NRBF2 complex further demonstrated that NSP4 occupies a central position spatially enveloped by NRBF2 residues (Figure 3J). This finding implies that NSP4 may initiate degradation via the autophagy pathway by recruiting NRBF2, thereby affecting the activation of the IFN‐I signaling pathway.

Figure 3NSP4 interacts with NRBF2. (A) Host proteins bound to NSP4 were removed using GST pull‐down and detected using LC‐MS/MS. (B) Differential host proteins fished. (C) NSP4‐host protein interaction network. (D) KEGG pathway enrichment. (E) Top 15 KEGG terms. (F) LLC‐PK1 cells were transfected with Flag‐tagged DNM2, AKT2, or NRBF2 plasmids and subsequently infected with RV for 24 h. Cells were then immunostained with an anti‐NSP4 antibody and an anti‐Flag antibody as primary antibodies, followed by fluorescently labeled secondary antibodies; nuclei were counterstained with DAPI. Scale bar, 10 μm. (G, H) Interaction of NSP4 with DNM2 and AKT‐2 was detected by Co‐IP in HEK293T cells. (H) Endogenous interaction between NSP4 and NRBF2 was examined in RV‐infected LLC‐PK1 cells. (I) Co‐IP analysis of the endogenous interaction between NSP4 and NRBF2 in RV‐infected LLC‐PK1 cells. (J) Prediction of NSP4 and NRBF2 protein structures using AlphaFold3 and modeling of their interactions using PyMOL.

**Figure:**
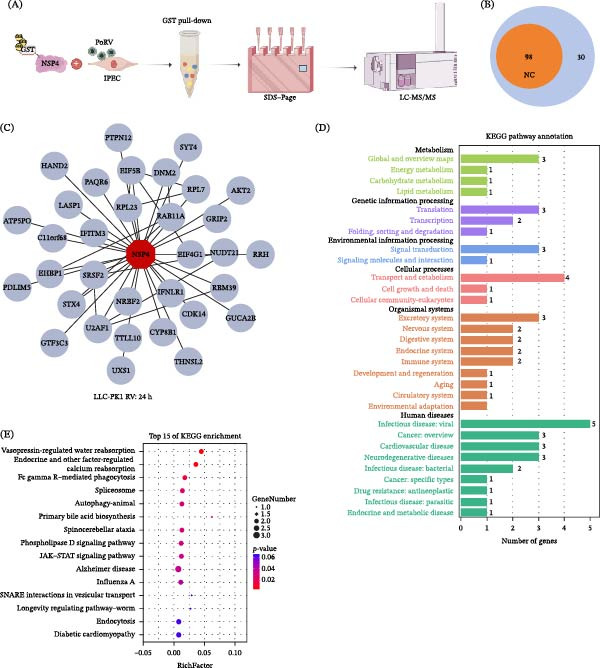

**Figure:**
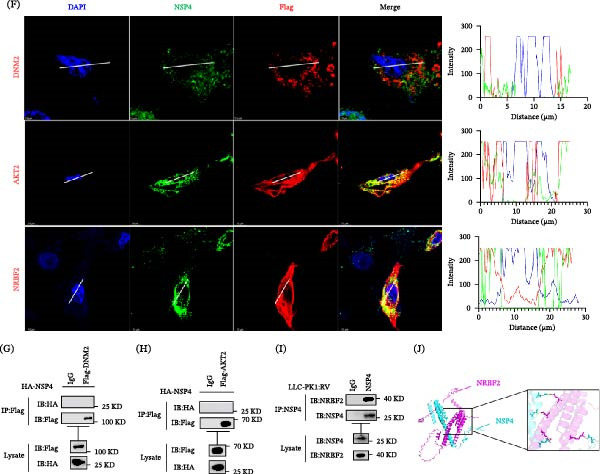

### 3.4. NRBF2 Degrades MDA‐5 Through the Autophagy Pathway

**Figure 4:**
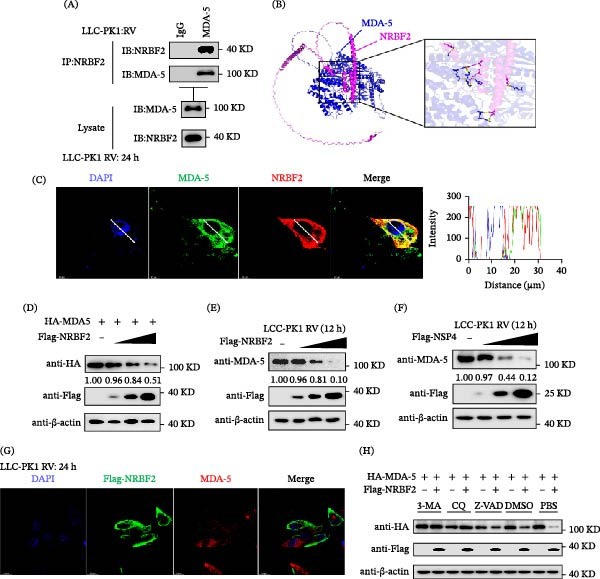
NRBF2 degrades MDA‐5 through the autophagy pathway. (A) Endogenous interaction between MDA5 and NRBF2 was examined in RV‐infected LLC‐PK1 cells. (B) AlphaFold3 was used to predict NRBF2 and MDA‐5 protein structures, and PyMOL was used to construct a model of their interaction. (C) LLC‐PK1 cells were infected with RV for 24 h, then immunostained with an anti‐MDA‐5 or anti‐NRBF2 antibody as the primary antibody followed by a fluorescently labeled secondary antibody; nuclei were counterstained with DAPI. Scale bar, 10 μm. (D) The corresponding plasmids were transfected into HEK293T cells, and the effects of NRBF2 on the stability of MDA‐5 were detected by Western blotting. (E, F) LLC‐PK1 cells were transfected with NSP4 and NRBF2 plasmids for 24 h and then infected with PoRV. The samples were collected after 12 h, and the effects of NRBF2 and NSP4 on the stability of MDA‐5 were examined by Western blotting with the corresponding endogenous antibodies. (G) LLC‐PK1 cells were transfected with Flag‐tagged NRBF2 plasmids and subsequently infected with RV for 24 h. Cells were then immunostained with an anti‐MDA‐5 antibody and an anti‐Flag antibody as primary antibodies, followed by fluorescently labeled secondary antibodies; nuclei were counterstained with DAPI. Scale bar, 10 μm. (H) The indicated plasmids were transfected into HEK293T cells for 18 h, which were then treated with 3‐MA (10 mM), CQ (20 mM), ZVAD (20 µM), DMSO, or PBS for 6 h and detected by Western blotting.

To investigate the impact of the NRBF2–NSP4 interaction on RLR signaling, Co‐IP assays confirmed an interaction between NRBF2 and MDA‐5 (Supporting Information 4: Figure S4A,B). We also examined the interaction between these two proteins during RV infection (Figure 4A). Structural modeling further revealed spatially intertwined configurations of MDA‐5 and NRBF2 (Figure 4B), while IIFAs confirmed their intracellular colocalization (Figure 4C). Western blot analysis of HEK293T cells demonstrated that NRBF2 triggered dose‐dependent degradation of MDA‐5, whereas RIG‐I and MAVS remained unaffected (Figure 4D and Supporting Information 4: Figure S4C,D). Consistent with these findings, endogenous MDA‐5 levels—but not RIG‐I or MAVS levels—were reduced in PoRV‐infected LLC‐PK1 cells at 12 h poststimulation, which was correlated with NRBF2 and NSP4 coexpression (Supporting Information 4: Figure S4E–H). Under RV infection conditions, NRBF2 indeed promotes the degradation of MDA‐5 (Figure 4G). To delineate the mechanism underlying MDA‐5 degradation, NRBF2 and MDA‐5 were coexpressed in the presence of the autophagy inhibitors 3‐MA and CQ. Both inhibitors restored the MDA‐5 protein level (Figure 4H), confirming that NRBF2 mediates MDA‐5 degradation via an autophagy‐dependent pathway. These findings collectively establish NRBF2 as a critical regulator of MDA‐5 stability, linking viral exploitation of the autophagic machinery to the suppression of innate immune signaling.

### 3.5. NSP4 Enhances NRBF2 Degradation of MDA‐5

**Figure 5:**
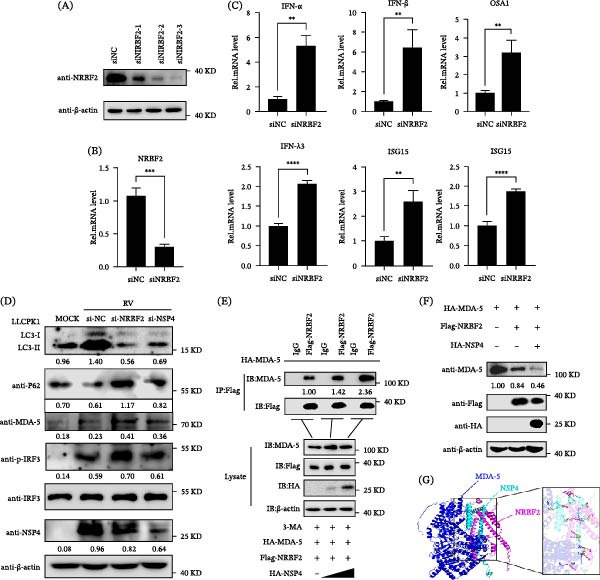
NSP4 enhances NRBF2 degradation of MDA‐5. (A) LLC‐PK1 cells were transfected with the indicated siRNAs, immunoblotted using an anti‐NRBF2 antibody as the primary antibody, and incubated with an HRP‐conjugated secondary antibody. (B) NRBF2 transcript levels were measured by RT‐PCR. (C) RT‐PCR was performed to assess the effects of NRBF2 knockdown on the transcript levels of the interferon‐stimulated genes IFN‐α, IFN‐β, OAS1, IFN‐λ, ISG15, and ISG56 in RV‐infected LLC‐PK1 cells. (D) Western blotting was performed to assess the effects of NRBF2 and NSP4 knockdown on autophagic flux, MDA‐5, and p‐IRF3 in RV‐infected LLC‐PK1 cells. (E) The effect of NSP4 on the interaction between MDA‐5 and NRBF2 was detected by Co‐IP in HEK293T cells. (F) The effects of the NSP4‐mediated regulation of NRBF2 on the stability of MDA‐5 were detected by Western blotting. (G) AlphaFold3 was used to predict the protein structures of NRBF2, NSP4, and MDA‐5, and PyMOL was used to construct a model of their interaction. *p* values less than 0.05 were considered statistically significant ( ^∗^ *p*  < 0.05,  ^∗∗^ *p*  < 0.01,  ^∗∗∗^ *p*  < 0.001, and  ^∗∗∗∗^ *p*  < 0.0001).

To validate the requirement of NRBF2 for the suppression of MDA‐5 and IFN‐I signaling, we knocked down NRBF2 and subsequently infected LLC‐PK1 cells with RV (Figure 5A,B). The results showed that NRBF2 silencing markedly enhanced the expression of ISGs and attenuated autophagic flux during viral infection. Consistently, the protein levels of MDA‐5 and p‐IRF3 were significantly increased (Figure 5C,D). To determine the role of NSP4 in the autophagic degradation of MDA‐5 by NRBF2, Co‐IP was performed in cells cotransfected with NRBF2 and MDA‐5, followed by dose‐dependent NSP4 supplementation and autophagy inhibition via 3‐MA. The results demonstrated that NSP4 potentiated the interaction between NRBF2 and MDA‐5 (Figure 5E). Consistently, coexpression of NRBF2, NSP4, and MDA‐5 revealed that NSP4 increased the degradation of MDA‐5 by NRBF2 without affecting RIG‐I or MAVS levels (Figure 5F and Supporting Information 5: Figure S5A,B). Structural modeling further revealed NSP4 as a spatial scaffold bridging MDA‐5 and NRBF2, facilitating their tight intermolecular associations (Figure 5I). Collectively, these data establish NSP4 as a critical facilitator of NRBF2‐mediated MDA‐5 degradation, orchestrating a ternary complex that primes MDA‐5 for autophagic clearance.

### 3.6. NSP4 Inhibits IFN‐I

To verify the ability of NSP4 to resist IFN‐I, we designed a siRNA to knockdown PoRV‐NSP4, detected changes in NSP4 transcript levels by qPCR (Figure 6A), and verified the knockdown efficiency in LLC‐PK1 cells (Figure 6B). Immunofluorescence experiments during PoRV infection revealed that the knockdown of NSP4 significantly increased the expression of MDA‐5, and the decrease in NSP4 expression increased the fluorescence value of MDA‐5 in the same field of view (Figure 6C). In addition, knockdown of PoRV‐NSP4 significantly attenuated viral infection (Figure 6D). Knockdown of PoRV‐NSP4 also significantly increased the transcript levels of the ISGs IFN‐α, IFN‐β, IFN‐λ, ISG15, ISG56, and OAS1 (Figure 6E). In addition, knockdown of NSP4 increased the phosphorylation levels of IRF3 during RV infection (Figure 6F), which further demonstrated that PoRV‐NSP4 inhibited the production of MDA‐5, affected the activation of ISGs as well as IRF3, and thus inhibited IFN‐I production. To simulate the role of PoRV‐NSP4 in PoRV infection, we isolated piglet intestinal organoids and cultured them in 3D to maturity (Figure 6G), digested them, and cultured them in 2D. On this basis, we also knocked down PoRV‐NSP4 and detected the transcript levels of ISG genes and IRF3 activation, and the results further demonstrated that PoRV‐NSP4 antagonized ISGs and IRF3 activation (Figure 6H,I) and inhibited IFN‐I production.

Figure 6NSP4 inhibits type I interferon. (A) RT‐PCR was used to detect changes in NSP4 transcript levels. (B) Changes in NSP4 protein levels in LLC‐PK1 cells were detected by Western blotting. (C, D) After NSP4 was knocked down in LLC‐PK1 cells and infected with RV for 24 h, the primary antibodies against MDA‐5, PoRV‐VP6, and PoRV‐NSP4 were used to stain the cells with fluorescent secondary antibodies, and the nuclei were stained with DAPI; scale bar, 20 μm. (E) RT‐PCR was performed to assess the effects of NSP4 knockdown on the transcript levels of the interferon‐stimulated genes IFN‐α, IFN‐β, IFN‐λ, ISG15, ISG56, and OAS1 in RV‐infected LLC‐PK1 cells. (F) Effect of NSP4 knockdown on p‐IRF3 stability was detected by Western blotting in RV‐infected LLC‐PK1 cells. (G) The process of intestinal organoid growth in vitro. (H) RT‐PCR was used to detect the effects of NSP4 knockdown on the transcript levels of the interferon‐stimulated genes IFN‐α, IFN‐β, IFN‐λ, ISG15, ISG56, and OAS1 in RV‐infected intestinal organoids cultured in 2D. (I) Effect of NSP4 knockout on p‐IRF3 stability in RV‐infected 2D intestinal organoids. All the experiments were independently repeated at least three times. The data are expressed as the means ± standard deviations; *n* = 3. *p* < 0.05 were considered statistically significant ( ^∗^ *p*  < 0.05,  ^∗∗^ *p*  < 0.01,  ^∗∗∗^ *p*  < 0.001, and  ^∗∗∗∗^ *p*  < 0.0001).

**Figure:**
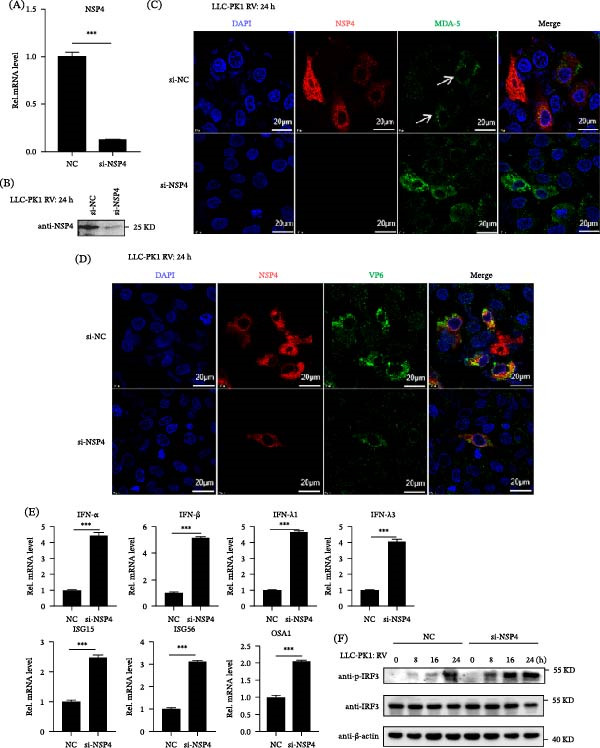

**Figure:**
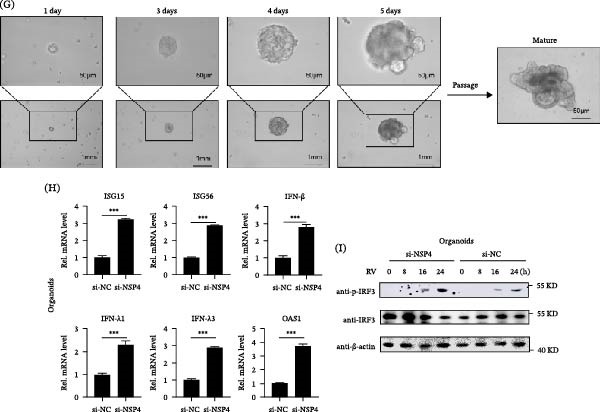

## 4. Discussion

PoRV is a leading cause of diarrheal disease in piglets, with mortality rates reaching 50%. As a zoonotic pathogen, it poses substantial threats to both global swine production and public health [2]. The innate immune system acts as the first line of antiviral defense, where virus‐triggered activation of the RLR signaling pathway induces IFN‐I production [26]. While earlier studies revealed that PoRV employs diverse mechanisms to antagonize host innate immunity, the role of its nonstructural protein NSP4 in immune evasion remains elusive. To address this gap, we investigated whether PoRV‐NSP4 facilitates immune escape by suppressing IFN‐I responses.

During viral infection, PRRs detect PAMPs, typically activating the RLR signaling pathway to induce IFN‐I production. IFN‐I binding to IFNAR1/2 initiates the expression of more than 300 ISGs, collectively suppressing viral replication and sustaining the host antiviral status [27]. Rotavirus nonstructural proteins (NSP1‐4) are exclusively expressed during infection. Emerging evidence reveals that these proteins facilitate immune evasion by subverting host surveillance and suppressing antiviral effector molecules [28]. Among them, NSP1 has been extensively characterized: it impedes STAT1 phosphorylation at Y701 to antagonize innate immune signaling and promotes the proteasomal degradation of IRF3, thereby inhibiting IFN‐I production. Notably, analogous to our findings, NSP1 targets RIG‐I, MDA‐5, and MAVS for degradation to block IFN‐I induction [29]. Numerous studies have shown that autophagy has numerous links with viral infection and host cells and that autophagy signaling pathways are involved in cellular homeostasis [30], developmental processes, cellular stress responses, and immunological pathways [31]. Numerous steps in the autophagy pathway can be used by a number of viruses [32], including rotavirus gastroenteritis virus [33], hepatitis C virus [34], and hepatitis B virus, to facilitate viral spread and immune response evasion [35]. For enterovirus infections, IFN‐III is induced to a greater extent than IFN‐I, but IFN‐Is are actually more potent against enteroviruses [36]. Recently, the mechanism by which IFN‐III signaling downregulates the expression of Dra, a chloride and bicarbonate exchanger that contributes to reduced water absorption and thus causes diarrhea, has been reported [37]. So, we are more convinced of the key antiviral role of IFN‐I in enterovirus infections.

In this study, we demonstrated that NSP4 mediates immune evasion by suppressing IFN‐I responses through MDA‐5 degradation via the autophagy pathway. Specifically, NSP4 recruits the autophagy‐related protein NRBF2 in a dose‐dependent manner to destabilize MDA‐5. Mechanistically, PoRV‐NSP4 acts as an adaptor to enhance the NRBF2‐MDA‐5 interaction, accelerating MDA‐5 turnover through autophagic degradation. These findings reveal a novel immune evasion strategy of PoRV that targets MDA‐5 to disrupt the IFN‐I signaling axis.

Importantly, NSP4 is also a known viroporin that perturbs ER calcium homeostasis, triggering cytosolic Ca^2+^ elevation [8, 38]. Given the central role of Ca^2+^ in regulating autophagy [39, 40], it is plausible that NSP4‐induced calcium dysregulation contributes to its ability to suppress IFN‐I signaling. In other words, the disruption of calcium homeostasis may prime the autophagic machinery, facilitating NRBF2‐mediated MDA‐5 degradation. This mechanism suggests that NSP4’s viroporin activity not only drives diarrheal phenotypes but also assists viral immune evasion. Future studies employing calcium channel inhibitors or modulation of intracellular Ca^2+^ levels, coupled with NRBF2/MDA‐5 analyses, could clarify the causal relationship between Ca^2+^ dysregulation and IFN‐I suppression, providing further mechanistic insights and potential antiviral targets.

Our findings establish that NSP4 employs multiple strategies to suppress IFN‐I‐mediated antiviral responses. Mechanistically, NSP4 not only inhibits the transcriptional activation of ISGs, IFN‐β promoter activity, and ISRE but also potently blocks p‐IRF3, a critical step for its nuclear translocation and subsequent IFN‐β induction. More critically, we identified a unique immune evasion mechanism in which NSP4 interacts with MDA‐5, recruiting the autophagy adaptor protein NRBF2 to promote MDA‐5 degradation via autophagy. This degradation event functionally disrupts RLR signaling and reduces host IFN‐I production. These discoveries increase our mechanistic understanding of how NSP4 not only exacerbates diarrhea by disrupting Ca^2+^ homeostasis but also orchestrates immune evasion during infection and illuminate the NSP4‐MDA‐5 axis as a compelling target for antiviral therapeutics and next‐generation vaccine development.

## 5. Conclusions

Our findings elucidate a previously unrecognized immune evasion mechanism by which NSP4 antagonizes the IFN‐I‐mediated antiviral response, providing novel molecular insights for developing therapeutic strategies against rotavirus infections.

## Author Contributions

**Jiaxing Zhan:** writing – reviewing and editing. **Tianhao Liang**: materials. **Jiale Chen**: maintain research data. **Jingying Wang**: data curation. **Enqi Dai**: investigation, methodology. **Ting Wang**: investigation, methodology. **Shaojie Wang**: data curation. **Zixuan Cao**: methodology. **Yan Zeng**: formulation or evolution of overarching research goals and aims. **Chunfeng Wang**: ideas. **Xin Cao**: design of methodology.

## Funding

This work was supported by the National Natural Science Foundation of China (Grants 32273043 and 32202890), the Science and Technology Development Program of Changchun City (Grant 21ZY42), and the Jilin Province Science and Technology Development Plan Item (Grant 20250102321JC).

## Disclosure

The authors have nothing to report.

## Ethics Statement

All experiments in this study were conducted according to the regulations of the Administration of Affairs Concerning Experimental Animals in China. The animal management procedures and all laboratory procedures abided by the regulations of the Animal Care and Ethics Committees of Jilin Agriculture University. The ethical review acceptance number is 20220302006.

## Conflicts of Interest

The authors declare no conflicts of interest.

## Supporting Information

Additional supporting information can be found online in the Supporting Information section.

## Supporting information


**Supporting Information 1** Figure S1: PoRV inhibits the activation of ISGs and IRF3. (A) LLC‐PK1 cells were infected with RV at different doses for 12 h, followed by SeV infection. The transcriptional levels of ISGs were then detected by qRT‐PCR. (B) PoRV infection of LLC‐PK1 cells activated p‐IRF3. (C) LLC‐PK1 cells were infected with RV at different doses for 12 h, followed by SeV infection. IRF3 phosphorylation was then analyzed by Western blotting.

**Supporting Information 2** Figure S2: NSP4 interacts with MDA‐5 and selectively reduces MDA‐5 protein levels without affecting RIG‐I or MAVS. (A) NSP4 interaction with MDA‐5 was detected by Co‐IP in HEK293T cells. (B) Endogenous interaction between NRBF2 and MDA‐5 was examined in RV‐infected LLC‐PK1 cells. (C, D) Effects of NSP4 on the stability of RIG‐I and MAVS were detected by Western blotting.

**Supporting Information 3** Figure S3: GST pull‐down screening and Co‐IP validation reveal the interaction between NSP4 and NRBF2. (A) Detection of the GST‐tagged NSP4 protein obtained from the construct using Western blotting. (B) SDS–PAGE detection of host proteins bound to NSP4. (C) Endogenous host proteins pulled down by NSP4 that were enriched in the animal autophagy pathway. (D) Interaction of NSP4 with DNM2, AKT‐2, and NRBF2 was detected by Co‐IP in HEK293T cells. (E) The interaction between NSP4 and NRBF2 was detected by coimmunoprecipitation in HEK293T cells.

**Supporting Information 4** Figure S4: NRBF2 interacts with MDA‐5 and selectively reduces MDA‐5 protein levels without affecting RIG‐I or MAVS. (A, B) NRBF2 and MDA‐5 signaling molecule interactions were detected by Co‐IP in HEK293T cells. (C, D) The corresponding plasmids were transfected into HEK293T cells, and the effects of NRBF2 on the stability of RIG‐I and MAVS were detected by Western blotting. (E–H) LLC‐PK1 cells were transfected with NSP4 and NRBF2 plasmids for 24 h and then infected with PoRV. The samples were collected after 12 h, and the effects of NRBF2 and NSP4 on the stability of MDA‐5 were examined by Western blotting with the corresponding endogenous antibodies.

**Supporting Information 5** Figure S5: NSP4 does not affect the stability of RIG‐I or MAVS in the presence of NRBF2. (A, B) The effects of the NSP4‐mediated regulation of NRBF2 on the stability of RIG‐I and MAVS were detected by Western blotting.

## Data Availability

The data that support the findings of this study are openly available at https://doi.org/10.6084/m9.figshare.29365313.v2 [41]. All uploaded data is ethically correct and does not contain any data related to the ethics, privacy, security, etc. of human subjects.
