# Supplementary figures and images for: Porcine Rotavirus NSP4 Inhibits Type I Interferon Production via NRBF2‐Mediated Autophagic Degradation of MDA‐5
Source: Transbound Emerg Dis. 2026 Jun 24;2026:5789277. doi: 10.1155/tbed/5789277 (PMC13291724; doi:10.1155/tbed/5789277)

## Slide 1
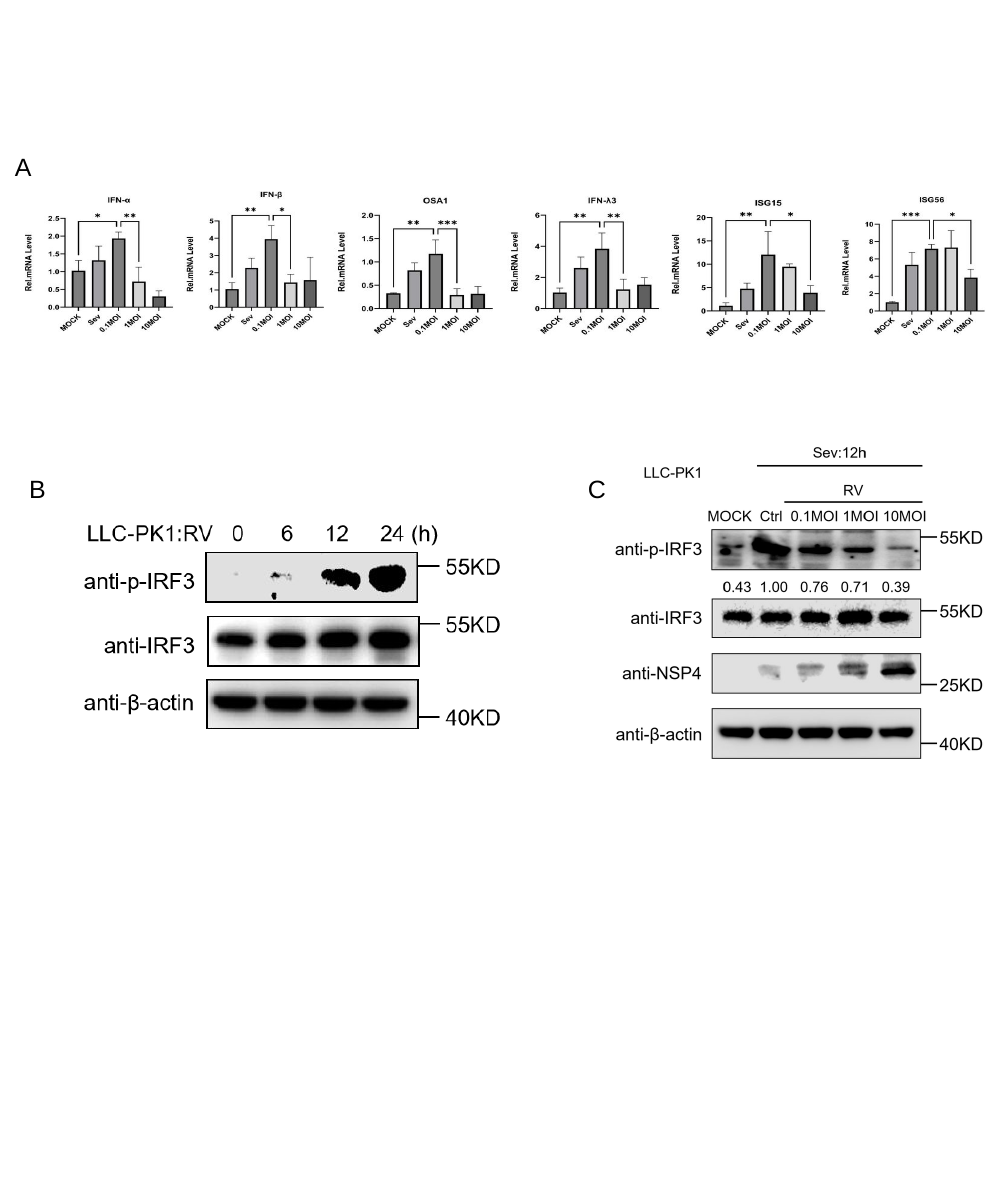

A
B
C

Supplement: Supplementary file 1 — Supporting Information 1 Figure S1: PoRV inhibits the activation of ISGs and IRF3. (A) LLC‐PK1 cells were infected with RV at different doses for 12 h, followed by SeV infection. The transcriptional levels of ISGs were then detected by qRT‐PCR. (B) PoRV infection of LLC‐PK1 cells activated p‐IRF3. (C) LLC‐PK1 cells were infected with RV at different doses for 12 h, followed by SeV infection. IRF3 phosphorylation was then analyzed by Western blotting. [file TBED-2026-5789277-s004.pptx]

## Slide 1
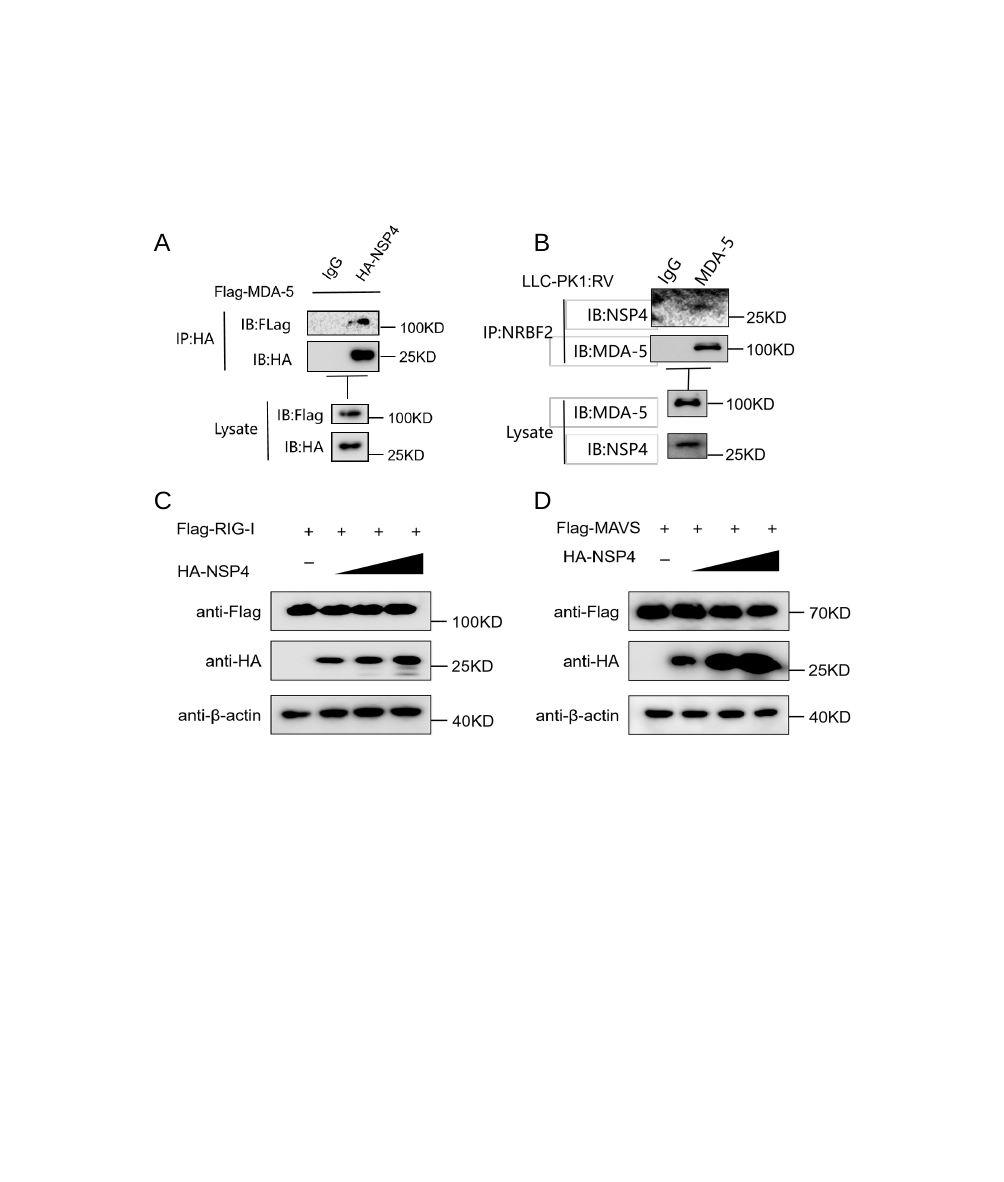

A
B
C
D

Supplement: Supplementary file 2 — Supporting Information 2 Figure S2: NSP4 interacts with MDA‐5 and selectively reduces MDA‐5 protein levels without affecting RIG‐I or MAVS. (A) NSP4 interaction with MDA‐5 was detected by Co‐IP in HEK293T cells. (B) Endogenous interaction between NRBF2 and MDA‐5 was examined in RV‐infected LLC‐PK1 cells. (C, D) Effects of NSP4 on the stability of RIG‐I and MAVS were detected by Western blotting. [file TBED-2026-5789277-s003.pptx]

## Slide 1
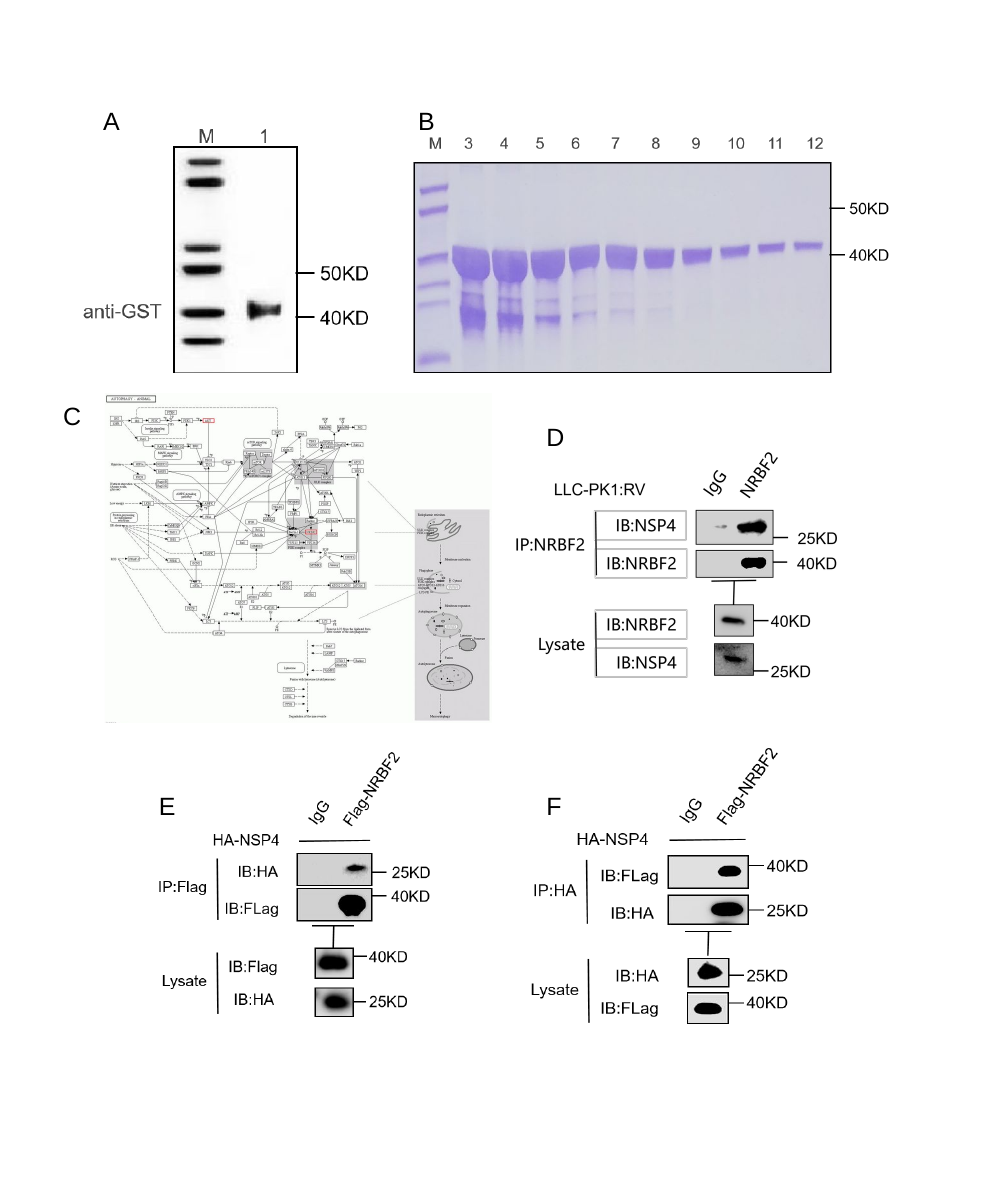

A
B
C
D
E
F

Supplement: Supplementary file 3 — Supporting Information 3 Figure S3: GST pull‐down screening and Co‐IP validation reveal the interaction between NSP4 and NRBF2. (A) Detection of the GST‐tagged NSP4 protein obtained from the construct using Western blotting. (B) SDS–PAGE detection of host proteins bound to NSP4. (C) Endogenous host proteins pulled down by NSP4 that were enriched in the animal autophagy pathway. (D) Interaction of NSP4 with DNM2, AKT‐2, and NRBF2 was detected by Co‐IP in HEK293T cells. (E) The interaction between NSP4 and NRBF2 was detected by coimmunoprecipitation in HEK293T cells. [file TBED-2026-5789277-s002.pptx]

## Slide 1
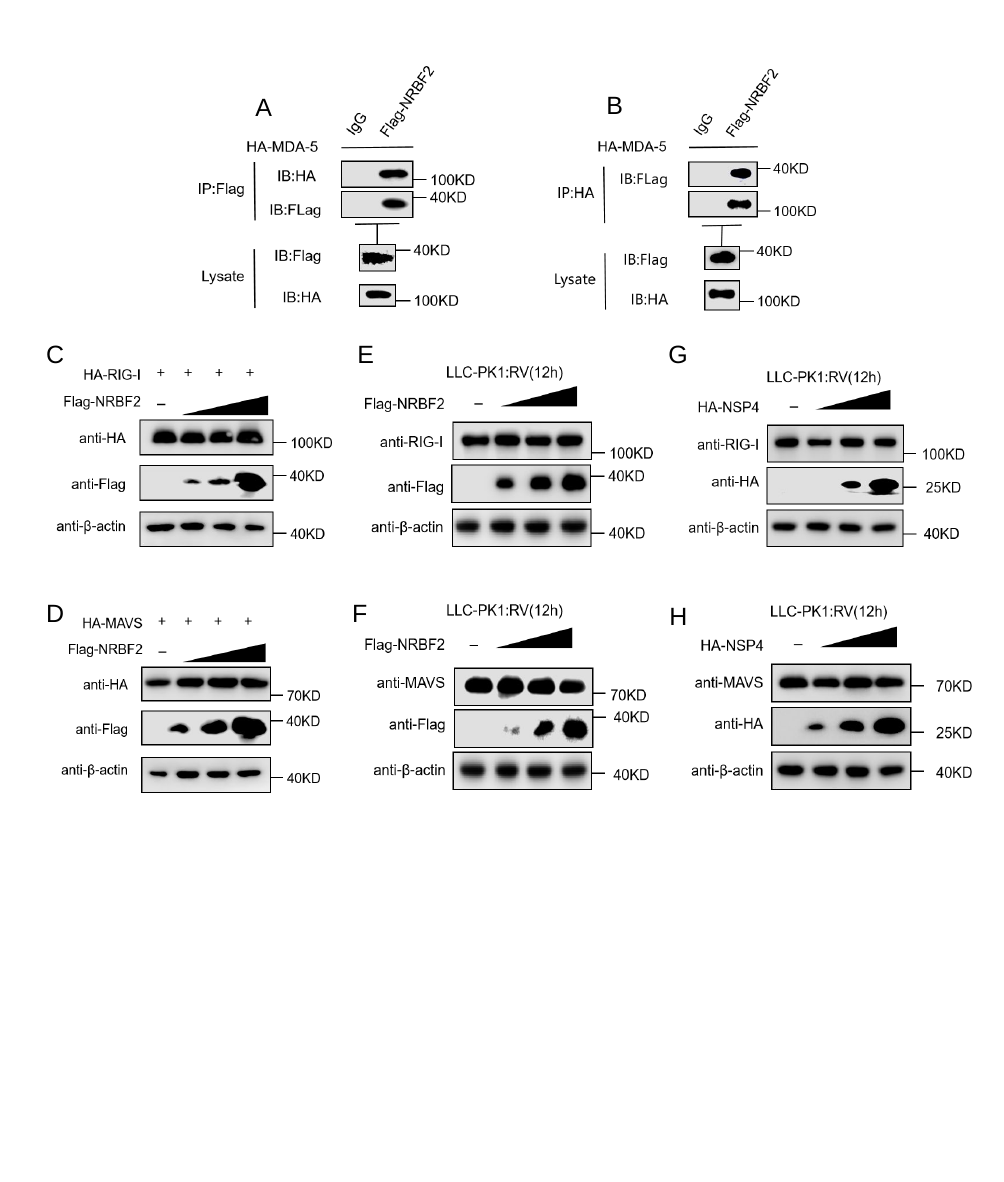

B
A
C
E
G
D
F
H

Supplement: Supplementary file 4 — Supporting Information 4 Figure S4: NRBF2 interacts with MDA‐5 and selectively reduces MDA‐5 protein levels without affecting RIG‐I or MAVS. (A, B) NRBF2 and MDA‐5 signaling molecule interactions were detected by Co‐IP in HEK293T cells. (C, D) The corresponding plasmids were transfected into HEK293T cells, and the effects of NRBF2 on the stability of RIG‐I and MAVS were detected by Western blotting. (E–H) LLC‐PK1 cells were transfected with NSP4 and NRBF2 plasmids for 24 h and then infected with PoRV. The samples were collected after 12 h, and the effects of NRBF2 and NSP4 on the stability of MDA‐5 were examined by Western blotting with the corresponding endogenous antibodies. [file TBED-2026-5789277-s001.pptx]

## Slide 1
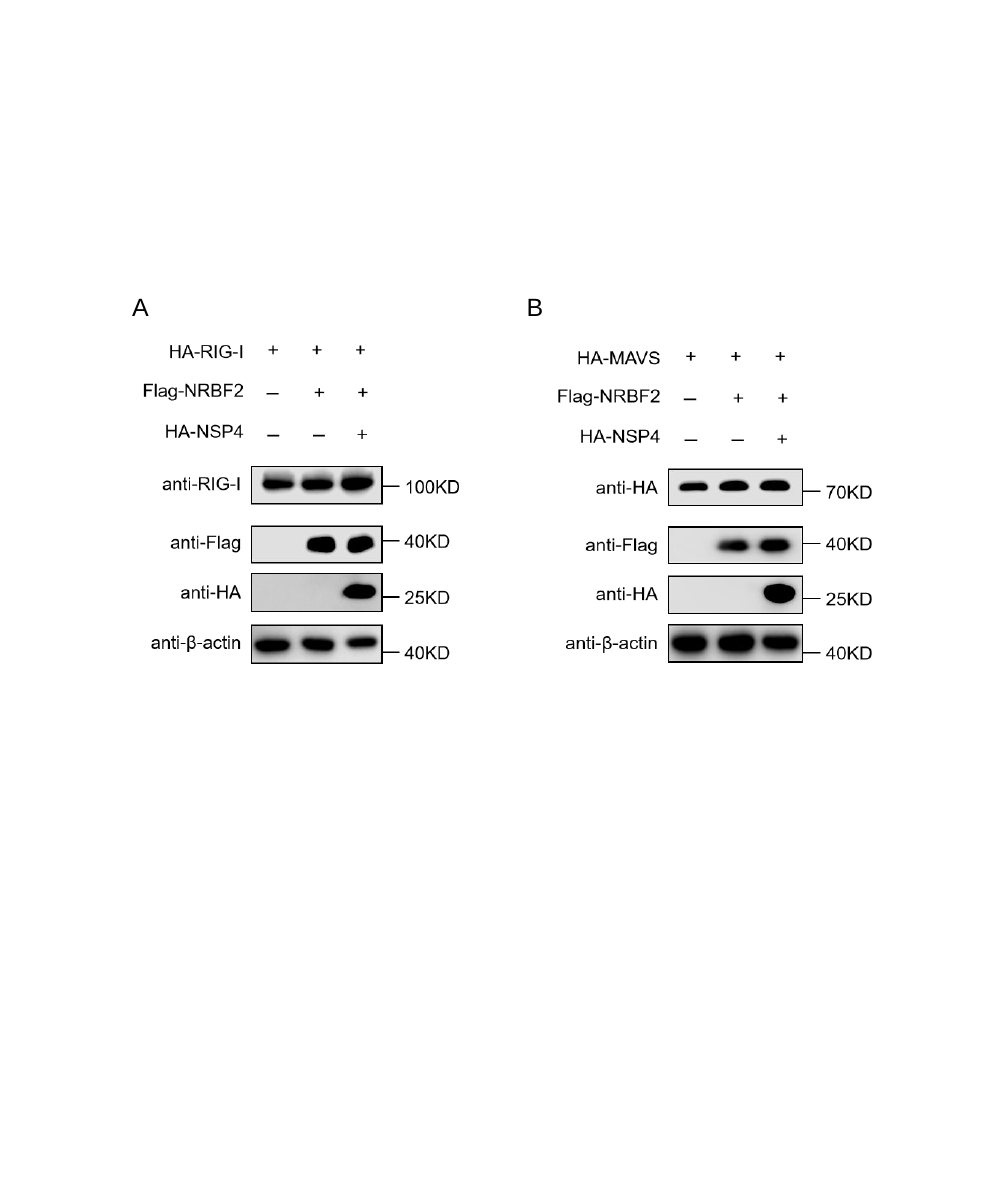

A
B

Supplement: Supplementary file 5 — Supporting Information 5 Figure S5: NSP4 does not affect the stability of RIG‐I or MAVS in the presence of NRBF2. (A, B) The effects of the NSP4‐mediated regulation of NRBF2 on the stability of RIG‐I and MAVS were detected by Western blotting. [file TBED-2026-5789277-s005.pptx]
